# Cardiac Diseases — Probable Atheromatous Deposits, Etc.

**Published:** 1873-03-01

**Authors:** N. S. Davis

**Affiliations:** Professor of Clinical Medicine, Etc.


### Clinical Lectures.

### CARDIAC DISEASES—PROBABLE ATHEROMATOUS DEPOSITS, ETC.

LECTURE DELIVERED IN MEDICAL WARDS OF MERCY HOSPITAL. BY N. S. DAVIS, M.D., PROFESSOR OF CLINICAL MEDICINE, ETC.

Gentlemen:—I bring to your notice to-day a case, the past history of which, with the present examination, will reveal several items of much interest and practical value. The patient before you is a man born in Europe; past the middle period of life; well-developed physical system; active, sanguine temperament; and a resident in this country only a few years. He first came under my observation one year since, when I found him occupying a bed in Ward 11 of this Hospital. At that time he had undergone some loss of flesh ; his face was pale, with a little œdematous puffiness under the eyes ; the skin was natural in temperature, and soft ; the pulse small, feeble, and extremely irregular ; the cardiac impulse feeble, and its motions so irregular and tremulous as to render it very difficult to analyze its sounds ; the respiration labored and difficult, with a harsh cough, and dry, wheezing, piping rhonchi over the whole chest, but with little or no dullness on percussion. His mouth and tongue were moist and clean ; urine scanty and high-colored; bowels slightly costive, and appetite poor. He had been gradually losing the use of his lower extremities for several months, and at the time of his admission into the Hospital, he could neither walk nor stand upon his feet, though he could move them a little by voluntary effort when lying in bed. The tops of his feet and ankles were slightly œdematous, and he complained of wandering, neuralgic pains. He had complained of shortness of breath, with palpitations, when exercising actively, and occasional attacks of asthma, for several months.

For these difficulties, some physician had made an issue in his left arm, near the insertion of the deltoid muscle. From some cause, this issue had assumed an appearance of gangrene, with an areola of dark redness and tumefaction around it.

Such was the condition of the patient at the time he came to the Hospital, about twelve months since. His occupation had kept him much within doors, and from early boyhood he had been accustomed to the use of fermented drinks and tobacco. His pipe was in use most of the time during the day; and so necessary was his drink considered, that his friends brought to the Hospital with him quite an array of bottles of what they called "*choice*" wine. Analysis showed no albumen in the urine; and after the action of the heart had been rendered more quiet and regular by rest and remedies, the only indication of valvular disease that could be detected was a slight purring or roughness accompanying the systole. In calling the attention of the clinical class, then attending the Hospital, to his case, the feeble cardiac impulse, with the constant tendency to irregular tremulus motion, and inefficient capillary circulation in the lungs, were attributed to fatty degeneration of the heart, aided by the more direct sedative effects of tobacco on the ganglionic and vaso-motor nervous apparatus. The same influences were also regarded as the efficient causes of the paraplegia and general muscular weakness. No one can understand the exact pathological conditions involved in this and similar cases, without a clear appreciation of the effects of certain habits on the processes of nutrition and disintegration or tissue metamorphosis.

Year after year this patient had kept his blood almost constantly impregnated with a small quantity of alcohol, by the daily use of wine and other fermented drinks. The presence of this agent not only lessened the daily interchange of carbonic acid gas for oxygen through the lungs, but also retarded the atomic changes in the tissues generally, and thereby favored the retention of an excess of effete matter, more especially carbonaceous matter, in the system. These deviations from the strictly healthy standard of action being slight, did not immediately develop notable morbid action, but from their persistence year after year they gradually caused an accumulation of fatty matter, most prominent in the omentum, giving prominence to the upper and anterior part of the abdomen, over the base of the heart, and finally the deposit of fat granules in the muscular structure of the heart and arteries. Similar changes frequently take place in the parenchymy of the liver and kidneys. In our patient the constant use of tobacco had added a still further debilitating influence on the functions of the pneumogastric and ganglionic nerves. Viewing his case in this aspect, I persuaded him at that time to wholly abandon the use of alcoholic beverages of every kind, and to limit the amount of tobacco as closely as possible.

To increase the capacity of the blood for absorbing oxygen in the lungs, and thereby increase the action of that agent on the fatty tissues, I gave the patient ten grains of chlorate of potassa, dissolved in mucilage of gum arabic, after each meal. To lessen the severity of his cough, and render his heart's action more steady and efficient, I gave him a teaspoonful of the following mixture before each meal-time:

℞.—Hydrochlorate of ammonia, ʒ iii.
Tart. ant. et. pot., 2 grs.
Sulph. morph., 3 grs.
Tinct. digitalis, ℥ i.
Syrup liquorice, ℥ iii.—Mix.

He was allowed any plain, light food his appetite might require. Under this management, in ten days, his circulation and respiration were so much improved, that the hydrochlorate of ammonia and digitalis mixture was limited to one dose before breakfast and at bed-time; but the chlorate of potassa was continued, and the thirtieth of a grain of strychnia added to each dose. At the end of three months he had so far recovered that he could walk with facility, and even go up and down stairs with only a little inconvenience on account of shortness of breath. He then nearly discontinued medical treatment and began to act in the capacity of a *nurse* for other patients in the Hospital, and has continued to act efficiently in that capacity until the present time. But during the last five or six weeks he has several times spoken to me about an increasing dyspnœa, especially on going up stairs, and occasional palpitation of the heart. On examining his chest by auscultation, a few days since, I found such morbid sounds as I thought would be interesting and profitable for you, and hence he has kindly come before you for examination to-day. If you will be both silent and patient, each one of you can take the stethoscope in turn, and listen carefully, over the cardiac region and up the course of the aorta to its arch; and you will find over the base of the heart, just on the median or sternal side of the nipple, a well marked, loud, valvular murmur, synchronous with the systole of the ventricles. As you move the stethoscope successively upward in the course of the aorta, the morbid sound rapidly diminishes until you reach the junction of the cartilage of the second rib with the sternum, and there you find a morbid sound, even more loud and rough than over the base of the heart. While making this examination you will also observe some mixture of dry and moist rhonchi, with prolonged expiration. The morbid sounds connected with the circulation, taken in connection with the previous history of the case, render it highly probable that the semi-lunar valves of the aorta have become thickened, and perhaps their edges fringed with atheromatous deposits, and that a similar change has taken place in the inner coats of the aorta, a little below its arch. The bronchial rhonchi are caused by chronic congestion, and perhaps thickening of the lining membrane of the smaller bronchial tubes. The ultimate prognosis is, of course, unfavorable; and yet by proper regulation of diet and exercise, avoiding all excesses, and as far as possible counteracting the tendency to atheromatous or fatty degenerations and deposits, by a return to the use of the chlorate of potassa dissolved in mucilage, he may still have life prolonged and rendered moderately useful for two or three years.

